# Repeated Superovulation Accelerates Primordial Follicle Activation and Atresia

**DOI:** 10.3390/cells12010092

**Published:** 2022-12-26

**Authors:** Qian Wang, Shu-Xian Zhao, Jian-Ning He, Hua Zhao, Bao-Xia Gu, Juan-Ke Xie, Yi-Jun Zhao, Cui-Lian Zhang, Zhao-Jia Ge

**Affiliations:** 1 Reproductive Medicine Center, People’s Hospital of Zhengzhou University, Henan Provincial People’s Hospital, Zhengzhou 450003, China; 2 College of Life Sciences, Institute of Reproductive Sciences, Key Laboratory of Animal Reproduction and Germplasm Enhancement in Universities of Shandong, Qingdao Agricultural University, Qingdao 266109, China; 3 College of Animal Science and Technology, Qingdao Agricultural University, Qingdao 266109, China

**Keywords:** mice, repeated superovulation, follicular development, mTORC1, PI3K

## Abstract

For humans, ARTs (assisted reproductive technologies) have become the most effective method to treat subfertility/infertility in clinic. To obtain enough oocytes during ART, ovarian stimulation is performed by exogenous hormones, and some patients undergo several ovarian stimulation cycles. Although some adverse effects of ARTs on women and offspring are reported, few studies are focused on the effects of multiple superovulation on ovarian reserve. In the present study, we found that repeated superovulation significantly reduced primordial follicle number and the serum AMH. Compared to the decreased antral follicle number, the expression of genes related to primordial follicle activation, such as *Foxo3*, *Akt*, and *Rptor,* and the atretic follicle number in ovaries were increased by superovulation times. We further found that repeated superovulation reduced the plasma level of FSH, LH, and estradiol, and increased the expression of genes related to apoptosis (*Bax*, *Casp3* (*caspase-3*), *Casp8*, and *Casp9*) in granulosa cells, providing evidence that repeated superovulation disrupted the balance between survival and death in granulosa cells. In summary, our results suggest that repeated superovulation has adverse effects on folliculogenesis.

## 1. Introduction

In human beings, assisted reproductive technologies (ARTs) have become the most effective way to treat infertility and subfertility in clinic. About 8 million children have been born via ARTs worldwide [1], and in some countries the incidence of ART births is up to 1–5% [2]. In human ART, one of the most important treatments is superovulation or controlled ovarian stimulation, which is generally performed using FSH (follicle-stimulating hormone) and the agonist or antagonist of gonadotropin-releasing hormone (GnRH) [3]. However, PMSG (pregnant mare serum gonadotropin) and hCG (human chorionic gonadotropin) are widely used for superovulation in mammals [4]. Some females would undergo more than one ovarian simulation cycle during reproductive age. It is reported that multiple superovulation has adverse effects on oocyte quality and embryonic development in mice [5,6,7,8], whereas the effects of repeated superovulation on ovarian reserve are not well elucidated. Dong et al. reported that repeated superovulation induced mitochondrial dysfunction in granulosa cells, but the incidence of follicles at different stages was not influenced [9] and the reproductive potential was not impaired in female rhesus monkeys [10]. Similar results about follicle development are also observed in the tammar wallaby [11]. Nevertheless, it is reported in mice that the ratios of primordial, primary, tertiary, and mature follicles are decreased if superovulated 4–6 times in mice [12], and premature ovarian failure can be induced by 5–15 consecutive superovulations [13]. In human beings, more ovarian stimulation also decreased the number of retrieved oocytes [14,15]. These suggest that repeated superovulation may have negative impacts on ovarian function, but the underlying mechanisms are still unknown. Furthermore, maintaining normal ovarian function is also crucial for long-term health. Premature ovarian insufficiency (POI) not only reduces female fertility years but also results in early amenorrhea, disrupted hypothalamic function, and low estrogen. Hypoestrogenemia increases the risk of cardiovascular diseases for premenopausal females [16,17]. Hypothalamic amenorrhea also induces bone resorption [18], higher cortisol level [19], and disrupted thyroidal function [20]. Therefore, it is crucial to elucidate the effects and mechanisms of repeated superovulation on follicular development.

## 2. Materials and Methods

### 2.1. Animals

Female ICR mice (5 weeks of age) were purchased from the Center of Experimental Animals of Qingdao and fed in a temperature- and humidity-controlled room at a cycle of 12 h light and 12 h darkness. Diet and water were supplied freely. All procedures were supported by the Animal Ethics Committee of Qingdao Agricultural University (QAU20190100175). Superovulation was performed as intraperitoneal injection with 8 IU PMSG (pregnant mare serum gonadotropin, Ningbo Hormone Product Co., Ltd., China) and 46–48 h later injected with 8 IU hCG (human chorionic gonadotropin, Ningbo Hormone Product Co., Ltd., Ningbo, China). Female ICR mice at 5 weeks of age were randomly divided into four groups: (a) non-superovulated group (R0); (b) superovulated one time (R1); (c) superovulated three times at a 7-day interval (R3); (d) superovulated five times at a 7-day interval (R5) [21]. To avoid the influence of stress induced by injection, R0, R1, and R3 were injected with saline 5, 4, and 2 times, respectively. Therefore, all mice underwent stimulation induced by injection 5 times and were at the same age when used.

### 2.2. Collection of Oocytes, Granulosa Cells, Plasma, and Ovaries

Mice were sacrificed by cervical dislocation. Cumulus–oocyte complexes were collected from oviduct ampulla at 13–14 h after hCG injection, and then cumulus cells were removed by incubation with 1% hyaluronidase in M2 medium [22]. After that, oocytes with and without first polar body were collected. For granulosa cells, ovaries were collected at 46–48 h after the injection of PMSG. Collected ovaries were put in M2 medium and grown follicles were pricked using a 1 mL syringe [23]. Then, granulosa cells were collected into tube and washed three times using PBS. A part of ovaries were stored at −80 °C or fixed with 4% PFA, immediately. While mice were sacrificed, blood was immediately collected from heart using a 1 mL syringe and transferred to an Eppendorf tube. After that, blood samples were clotted at 4 °C for 2 h, and then centrifuged at 704× *g* for 10 min to harvest serum. Serum samples were stored at −80 °C until use.

### 2.3. ELISA

Serum level of AMH (Anti-Müllerian hormone), LH (luteinizing hormone), FSH (follicle-stimulating hormone), and estradiol was examined using ELISA kits (Sangon Biotech, Shanghai, China) according to the handbook. Briefly, standard and samples were added into relative plate, and then covered with sealers. After that, it was incubated at 37 °C for 30 min. Then HRP-conjugated reagent was added and incubated for 30 min at 37 °C. After the treatment of TMB substrate A & B solution, reaction was stopped and the absorbance was examined using Multiscan Spectrum. The standard curve was generated by four-parameter logistic fit using software.

### 2.4. H & E Staining (Hematoxylin and Eosin Staining) and Follicle Counting

To calculate the number of follicles, ovaries were fixed with 4% PFA overnight, dehydrated with ethanol, treated with dimethylbenzene and embedded in paraffin. Embedded ovaries were sectioned at 5 µm thickness. Sections were distributed on glass slides. After that, sections were dewaxed with xylene, dehydrated with 100%, 95%, 85%, 70%, and 50% ethanol, and then stained with hematoxylin and eosin. The number of different stage follicles was calculated as previously reported [24]. Briefly, follicles were counted every fifth section under microscope, and the total follicle number of 5 slides was used to present the follicles per ovary. To avoid repeated counts of the same follicle, only follicles with visible oocyte nucleus were included [13]. Follicles were classified as: primordial follicle, one layer of flattened granulosa cells surrounding the oocyte; primary follicle, one to two complete layers of cuboidal granulosa cells; secondary follicle, an oocyte surrounded by more than one layer of cuboidal granulosa cells with no visible antrum; antral follicle, an oocyte surrounded by multiple layers of cuboidal granulosa cells and containing antral spaces, possibly with a cumulus oophorus and thecal layer; and atretic follicle, a follicle that enters a degenerative process without ovulation [25].

### 2.5. Total RNA Purification and Relative Quantitative Real-Time PCR (RT-qPCR)

Total RNA of granulosa cells and ovaries were purified using miRNeasy Micro Kit (Qiagen) according to the manufacturer’s protocol. Briefly, granulosa cells or homogenized ovaries were lysed with Lysis reagent. Then, chloroform was added. After centrifugation, the upper aqueous phase was transferred to a new tube and 1.5 volumes 100% ethanol was added. After that, total RNA was purified using spin column.

The synthesis of the first strand cDNA of total RNA was performed using 1st Strand cDNA Synthesis SuperMix (Yeasen Biotechnology, Shanghai, China) according to the manufacturer’s protocol. RT-qPCR was carried out using ABI machine (ABI), and *Ppia* and *Gapdh* were used as reference [26]. Briefly, total volume for each reaction is 20 µL including 0.5 µL primers, 10 µL SYBR Green (Vazyme, Shanghai, China), 2 µL cDNA and water. Three replicates were run for each sample, and 40 cycles at 94 °C for 15 s (second), 60 °C for 15 s, and 72 °C for 30 s were performed. After that, if the melt curve had a single peak at about 85 °C, the relative expression was calculated by 2^−ΔΔCt^ [27]. Primers were obtained from Primer Bank (Table 1).

### 2.6. Statistical Analysis

Data were presented as mean ± SD, and the statistical difference was calculated by Student *t*-test (Unpaired *t*-test) using GraphPad Prism [28]. If *p*-value < 0.05, the difference was recognized as significant.

## 3. Results

### 3.1. Repeated Superovulation Reduces the Oocyte Number

**Figure 1:**
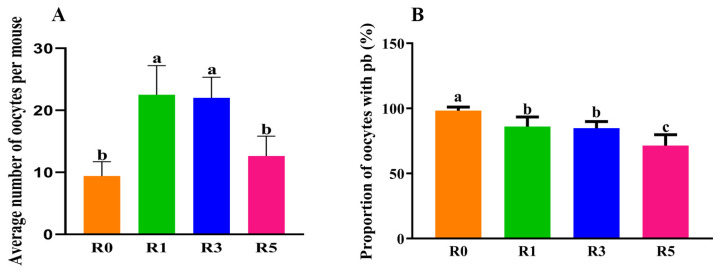
Repeated superovulation changed oocyte number. (**A**), with the increase in superovulation time, the average number of ovulated oocytes was decreased; each dot represents the average oocytes per mouse in each repeated experiment; (**B**), the ratio of oocytes with 1st polar body was also analyzed; each dot represents the pb proportion of ovulated oocytes in each repeated experiment. Repeated superovulation reduced ratio of mature oocytes. The same letters mean the statistical difference was not significant between groups (*p* > 0.05, unpaired *t*-test); different letters mean the statistical difference was significant between groups (*p* < 0.05, unpaired *t*-test).

In the present study, we investigated the influence of repeated superovulation on oocytes in mice. Compared to R0 (repeated 5 times, *n* = 50), the average number of ovulated oocytes was significantly higher in R1 (repeated 6 times, *n* = 60, *p* < 0.05) and R3 (repeated 6 times, *n* = 60). However, if superovulation cycles were up to 5 times (repeated 8 times, *n* = 80, *p* < 0.05), the average ovulated oocyte number was slightly higher than that in R0 (Figure 1A) and significantly lower compared with R1 and R3. We also found that the rate of ovulated oocytes with 1st bp (first polar body) was reduced in R1 (*n* = 184 mice, *p* < 0.05), R3 (*n* = 206 mice, *p* < 0.05), and R5 (*n* = 385 mice, *p* < 0.05) compared with R0 (*n* = 291 mice) (Figure 1B). This suggests that multiple superovulations have an adverse influence on oocyte maturation in mice.

### 3.2. Follicular Development Is Impaired by Repeated Superovulation

**Figure 2:**
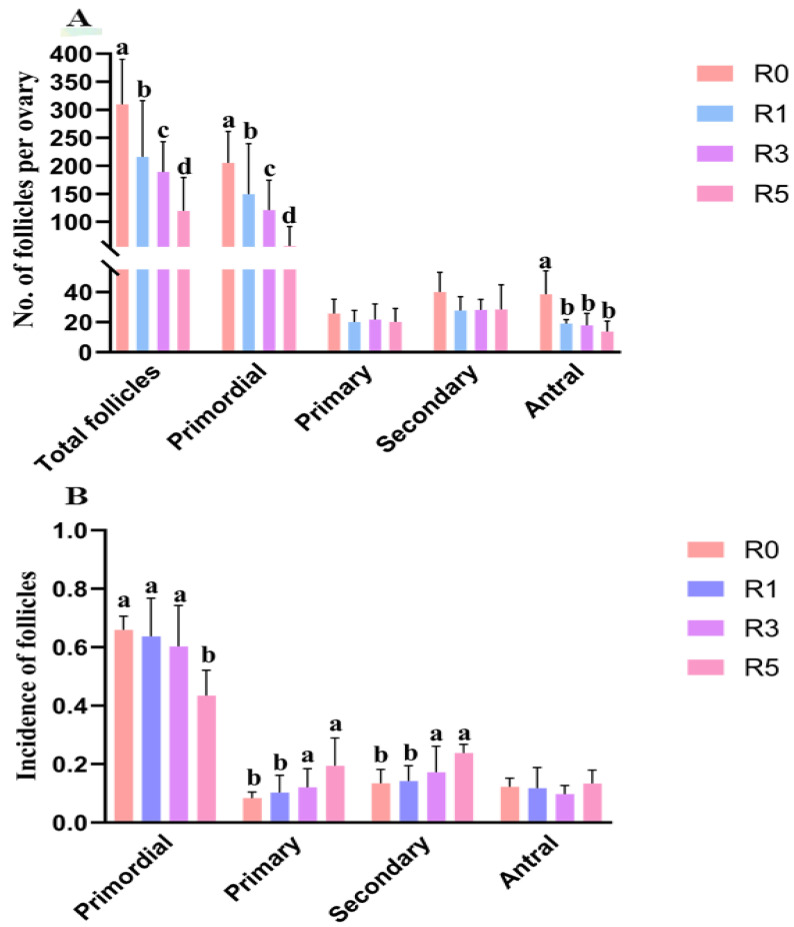
Follicles in ovaries. (**A**), the number of different stage follicles in ovaries; (**B**), the ratios of follicles at different stages. The same letters mean the statistical difference was not significant between groups (*p* > 0.05, unpaired *t*-test); different letters mean the statistical difference was significant between groups (*p* < 0.05, unpaired *t*-test).

To further explore the impact of multiple superovulation on ovarian function, we analyzed follicular development in ovaries (*n* = 6 for each group). We found that, with the increase in superovulation times, the total follicle number was significantly decreased in ovaries compared with R0 (Figure 2A). Compared with R0, the number of primordial follicles was significantly reduced in R1, R3, and R5 (Figure 2A, *p* < 0.05). The number of primordial follicles in R5 was also significantly lower than that in R1 and R3 (Figure 2A), whereas the number of primary and secondary follicles was similar in the groups. The antral follicle number in R0 was higher than that in R1, R3, and R5. Although there was a declining trend in the number of antral follicles with the increase in superovulation times, the statistical difference was not significant in R1, R3, and R5 (Figure 2A).

As mentioned, the total number of follicles was reduced by superovulation; thus, we analyzed the effects of repeated superovulation on ratios of follicles at different stages to further investigate the effects of repeated superovulation on follicles. The results showed that the incidence of primordial follicles was similar between R0, R1, and R3, but it was significantly lower in R5 compared with R0, R1, and R3. The ratios of primary and secondary follicles were increased in R5 compared with R0. The percentage of antral follicles was similar in the groups (Figure 2B). These suggest that repeated superovulation might accelerate the activation of primordial follicles and apoptosis of developing follicles.

### 3.3. Changes in Plasma Hormone Level May Be Involved in the Abnormality of Follicular Development

**Figure 3:**
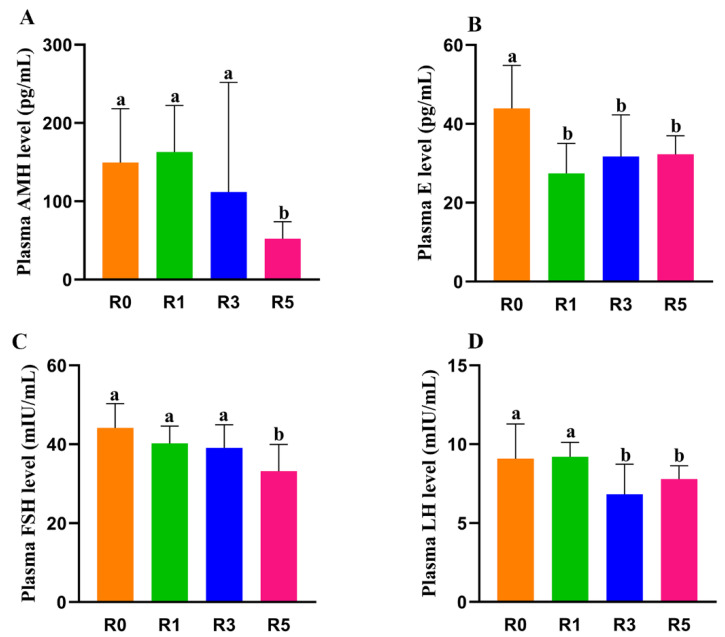
Plasma concentration of hormones. The plasma concentration of hormones was examined by ELISA. (**A**), AMH; (**B**), E (estradiol); (**C**), FSH; and (**D**), LH. The same letters mean the statistical difference was not significant between groups (*p* > 0.05, unpaired *t*-test); different letters mean the statistical difference was significant between groups (*p* < 0.05, unpaired *t*-test).

AMH is secreted by granulosa cells and is essential to inhibit primordial follicle activation. We found that repeated superovulation significantly decreased serum AMH level (Figure 3A). The AMH level in R1 was similar to R0, but it was lower in R3 and R5. The AMH level in R5 was also lower than that in R3. This suggests that the reduced AMH level induced by repeated superovulation may play a role in the decrease in primordial follicles. In this study, we also found that the plasma estrogen level in R0 was higher than that in R1, R3, and R5, but there was no significant difference in R1, R3, and R5 (Figure 3B). Considering the important role of FSH and LH in folliculogenesis, the serum level of FSH and LH (*n* = 10–12 for each group) was further tested and we found that serum FSH and LH decreased with the increase in superovulation times (Figure 3C,D). These results indicate that the abnormal follicular development caused by multi-superovulation may be associated with the decreased level of relative hormones.

### 3.4. Repeated Superovulation May Accelerate Primordial Follicle Activation via PI3K-PTEN-AKT-FOXO3 and mTORC1 Signaling

**Figure 4:**
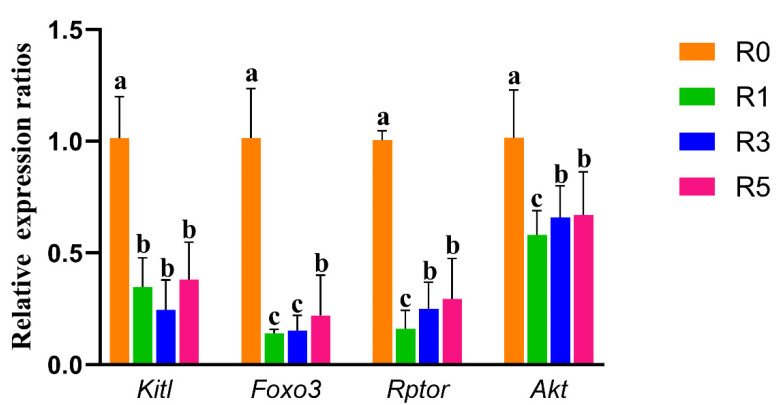
The expression of genes related to primordial follicle activation. The expression of *Rptor*, *Akt*, *Foxo3*, and *Kitl* in ovaries was analyzed by RT-qPCR. *Ppia* and *Gapdh* were used as reference. The relative expression was calculated by 2^-ΔΔCt^. The same letters mean the statistical difference was not significant between groups (*p* > 0.05, unpaired *t*-test); different letters mean the statistical difference was significant between groups (*p* < 0.05, unpaired *t*-test).

**Figure 5:**
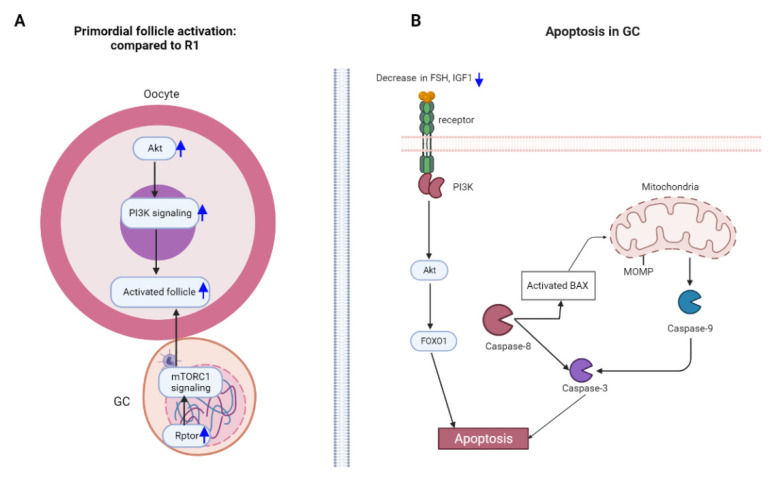
Schedule of repeated superovulation regulating primordial follicle activation and granulosa cell apoptosis. (**A**), schedule of regulating primordial follicle activation by repeated superovulation; (**B**), schedule of granulosa cell apoptosis. GCs—granulosa cells; R1—superovulated 1 time.

Primordial follicle activation is regulated by the communication between primordial follicle granulosa cells and oocytes via mTORC1 and PI3K-PTEN-AKT-FOXO3 signaling. In the present study, we tested the expression of *Rptor* (regulatory-associated protein of MTOR, complex 1) and *Kitl* (Kit ligand) in ovaries (*n* = 4 for each group) and found that the expression of *Rptor* and *Kitl* was lower in ovaries of R1, R3, and R5 compared with R0 (Figure 4). However, compared with R1, the expression of *Rptor* was significantly increased in R5. There was a slight increase in the expression of *Kitl* in R5 compared with R1. We also observed decreased expressions of *Akt* and *Foxo3* in R1, R3, and R5 compared with R0 (Figure 4). Compared with R1, the expression of *Akt* in R5 was significantly increased (Figure 4). These results suggest that the mTORC1 and PI3K-PTEN-AKT-FOXO3 pathways might be involved in the accelerated awakening of primordial follicles induced by repeated superovulation (Figure 5A).

### 3.5. Granulosa Cell Apoptosis Induced by Repeated Superovulation May Play a Role in the Abnormality of Follicular Development

**Figure 6:**
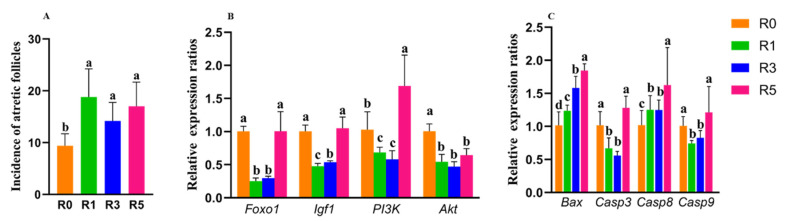
Effects of repeated superovulation on granulosa cell apoptosis. (**A**), average number of follicular atresia in ovaries; (**B**), the expression of genes related to anti-apoptosis in granulosa cells; (**C**), the expression of genes related to apoptosis in granulosa cells. The same letters mean the statistical difference was not significant between groups (*p* > 0.05, unpaired *t*-test); different letters mean the statistical difference was significant between groups (*p* < 0.05, unpaired *t*-test).

As shown in Figure 1A and Figure 2A, the increase in superovulation times reduces the number of ovulated oocytes and antral follicles. These indicate that repeated superovulation may increase follicular atresia. Then, the number of atretic follicles was detected and we found that repeated superovulation significantly increased the number of atretic follicles (Figure 6A). To further elucidate the possible mechanisms, we examined the expression of survival factors such as *Foxo1*, *Igf1*, *Akt*, and *PI3K* in granulosa cells (*n* = 5–6 for each group). We found that, compared with R0, the expression of *Foxo1*, *Igf1*, *Akt*, and *PI3K* was significantly decreased in R1 and R3 (Figure 6B). However, the expression of *Foxo1*, *Igf1*, and *PI3K* in R5 was similar or higher than that in R0. Compared with R1 and R3, the expression of *Foxo1*, *Igf1*, *Akt*, and *PI3K* was significantly higher in R5 (Figure 6B). This indicates that, although superovulation was adverse to granulosa cells’ survival, with the increase in superovulation times, a possible survival mechanism might be activated in granulosa cells to prevent death.

We further investigated the expression of apoptotic genes in granulosa cells, such as *Bax*, *Casp3* (Caspase3), *Casp8*, and *Casp9*. Compared with R0, superovulation increased the expression of *Bax* and *Casp8* (Figure 6C). In R5, the expression of *Bax*, *Casp3*, *Casp8*, and *Casp9* was significantly higher than that in R1 and R3 (Figure 6C). These results suggest that multiple superovulation disrupted the balance between death and survival in granulosa cells, which may mediate the follicular atresia (Figure 5B).

## 4. Discussion

In the present study, we found that repeated superovulation: (1) decreased the number of primordial follicles and antral follicles; altered (2) the plasma levels of AMH, estradiol, LH, FSH; and (3) the expression of genes involved in primordial follicle activation in ovaries, such as *Rptor, Kitl, Foxo3,* and *Akt*; and (4) the expression of genes involved in granulosa cell survival, such as *Foxo1, Igf1, Akt,* and *PI3K*; and (5) increased the expression of genes relative to apoptosis in granulosa cells, such as *Bax, Casp3, Casp8,* and *Casp9*.

At birth, oocytes are arrested at the diplotene phase of meiosis I and form primordial follicles, and, at puberty, the activated primordial follicles can develop to mature oocytes [29]. The activation of primordial follicles is complex. It is reported that AMH secreted by granulosa cells of growing follicles is relative to primordial follicle activation [30]. Knockout AMH accelerates primordial follicle activation and leads to premature ovarian failure in mice [31]. Exogenous AMH application protects the ovarian reserve [32]. The expression of AMH is regulated by estradiol (E) [33], and deletion of the estrogen receptor decreases AMH level and increases the activation of AKT and mTORC1 signaling [34]. Therefore, the decreased estradiol level induced by superovulation may play a role in decreasing the plasma level of AMH, which may be a reason for the reduction in primordial follicles induced by repeated superovulation.

Primordial follicle activation is also regulated by the communication between the primordial follicle granulosa cells (pfGCs) and oocytes via the mTORC1 and PI3K-PTEN-AKT-FOXO3 pathways [35]. Zhang et al. reported that the deletion of *Rptor*, the regulatory-associated protein of mTOR, inhibits the mTORC1 signaling in pfGCs, which prevents dormant oocytes’ activation. Active mTORC1 signaling increases the expression of *Kitl*, leading to the premature awakening of primordial follicles via activating the PI3K pathway in oocytes [36]. In primordial oocytes, active *Foxo3* (Forkhead box O3) inhibits primordial follicle activation in mice. However, the activation of *Foxo3* can be inhibited by *Akt* (thymoma viral proto-oncogene 1) which can be activated via the PI3K pathway [37]. We found that superovulation decreased the expression of *Foxo3* in ovaries and, compared with R1, the expression of *Rptor* and *Akt* was higher in R5. These results suggest that repeated superovulation may accelerate primordial follicle activation via the mTORC1 and PI3K-PTEN-AKT-FOXO3 pathways.

Follicular development is a complex process including morphological and functional changes of granulosa and theca cells. Gonadotropin plays a key role in this process and regulates follicular development by the two-cell two-gonadotropin system. FSH is responsible for preantral and antral follicular development via regulating granulosa cell proliferation, the synthesis of estrogen during follicular development [38,39], the expression of LH receptor in granulosa cells [40], which is mediated by estrogen [41], and so on. A deficiency of FSH induces the expression of apoptotic factors and follicular atresia [42]. For preovulatory follicles, LH stimulates theca cells to synthesize androgen, which is converted into estrogen in granulosa cells [41,43,44]. Estrogen increases the secretion of gonadotropin that regulates ovulation, oocyte maturation, and luteinization [45,46]. In the present study, we found that repeated superovulation decreased the serum level of FSH, LH, and estradiol, which might play a role in abnormal follicular development.

Follicular atresia is mediated by granulosa cell apoptosis, which is regulated by death and survival pathways [47]. It has been demonstrated that FSH, estradiol, and IGF1 are crucial prosurvival factors of granulosa cells [47]. Anti-apoptosis of these hormones in granulosa cells is mediated by PI3K-AKT signaling [48]. FSH and IGF1 activate PI3K and AKT, which enhance the expression of *Foxo1* and phosphorylation [49,50]. The phosphorylated FOXO1 inhibits the expression of proapoptotic factors, such as *Bax* in granulosa cells [51,52]. We found that superovulation reduced the expression of *Foxo1*, *Igf1*, *Akt*, and *PI3K*, and increased the expression of *Bax* in granulosa cells. These indicate that the decreased hormones may enhance granulosa cell apoptosis via the PI3K-AKT pathway.

However, it is interesting that, compared with R1 and R3, the expression of *Igf1*, *Akt*, *PI3K*, and *Foxo1* was higher in granulosa cells of R5. However, if superovulation is more than 5 times, the expression of *Sirt1* and *Foxo1* is clearly reduced [13]. This suggests that the incidence of different stage follicles might not be significantly decreased if superovulation is less than 5 times. Liu et al. showed that the ratios of follicles at different stages were clearly changed if superovulation is 4–6 times [12]. Three times superovulation has no effects on the incidence of different stage follicles in the tammar wallaby [11]. In rhesus monkeys, four cycles of ovarian stimulation do not change the ratios of different stage follicles in ovaries [9]. In the present study, we found that repeated superovulation has no significant effect on the incidence of antral follicles, but increased the ratios of primary and secondary follicles in R3 and R5.

Besides survival factors, apoptotic factors are also important for granulosa cell apoptosis [47]. In granulosa cells, *Casp8* can activate the mitochondrial apoptotic pathway by stimulating the expression of *Bax* and *Casp9*, and cytochrome c release, which further induces apoptosis via *Casp3* [53]. *Casp8* also can directly activate *Casp3* to induce apoptosis in granulosa cells [52,54]. In the present study, we found that repeated superovulation increased the expression of *Bax*, *Casp3*, *Casp8*, and *Casp9* in granulosa cells. This suggests that the increased expression of apoptotic factors may play a crucial role in granulosa cell apoptosis, leading to a reduction in follicle numbers in ovaries, and granulosa cell apoptosis may be mediated mainly by the apoptotic pathway.

In summary, the decreased AMH and abnormal expression of genes related to the mTORC1 and PI3K-PTEN-AKT-FOXO3 pathways might be involved in accelerating primordial follicle activation induced by repeated superovulation. In addition, the decreased number of antral follicles may be regulated by the imbalance between death and survival in granulosa cells. However, more studies are necessary to elucidate the underlying mechanisms of repeated superovulation regulating folliculogenesis in the future. It is also important to explore effective ways to reduce the negative effects of repeated superovulation on ovarian function. The present study was conducted with mice, and more evidence is required to elucidate the effects of multiple ovarian stimulations on humans.

## Figures and Tables

**Table 1:** Primers for RT-qPCR.

Genes	Sequence
*Casp9-F*	TCCTGGTACATCGAGACCTTG
*Casp9-R*	AAGTCCCTTTCGCAGAAACAG
*Casp8-F*	TGCTTGGACTACATCCCACAC
*Casp8-R*	TGCAGTCTAGGAAGTTGACCA
*Casp3-F*	TGGTGATGAAGGGGTCATTTATG
*Casp3-R*	TTCGGCTTTCCAGTCAGACTC
*Bax-F*	TGAAGACAGGGGCCTTTTTG
*Bax-R*	AATTCGCCGGAGACACTCG
*Igf1-F*	GTGAGCCAAAGACACACCCA
*Igf1-R*	ACCTCTGATTTTCCGAGTTGC
*PI3k-F*	ACACCACGGTTTGGACTATGG
*PI3k-R*	GGCTACAGTAGTGGGCTTGG
*Akt-F*	AGAAGAGACGATGGACTTCCG
*Akt-R*	TCAAACTCGTTCATGGTCACAC
*Foxo1-F*	GGGTCCCACAGCAACGATG
*Foxo1-R*	CACCAGGGAATGCACGTCC
*Foxo3-F*	CTGGGGGAACCTGTCCTATG
*Foxo3-R*	TCATTCTGAACGCGCATGAAG
*Kitl-F*	GAATCTCCGAAGAGGCCAGAA
*Kitl-R*	GCTGCAACAGGGGGTAACAT
*Rptor-F*	TTTGTCTACGACTGTTCCAATGC
*Rptor-R*	GCTACCTCTAGTTCCTGCTCC

Note: F, forward primer; R, reverse primer.

## Data Availability

All data generated or analyzed during this study are included in this published article.
